# Health care providers’ experiences of pain management and attitudes towards digitally supported self-management interventions for chronic pain: a qualitative study

**DOI:** 10.1186/s12913-021-06278-7

**Published:** 2021-03-25

**Authors:** Cecilie Varsi, Ingrid Konstanse Ledel Solem, Hilde Eide, Elin Børøsund, Olöf B. Kristjansdottir, Karina Heldal, Lori B. Waxenberg, Karen E. Weiss, Karlein M. G. Schreurs, Eleshia J. Morrison, Audun Stubhaug, Lise Solberg Nes

**Affiliations:** 1 Department of Digital Health Research, Division of Medicine, Oslo University Hospital, Pb 4950 Nydalen, N-0424 Oslo, Norway; 2 Faculty of Health and Social Sciences, University of South-Eastern Norway, Drammen, Norway; 3 Institute of Clinical Medicine, University of Oslo, Oslo, Norway; 4 Science Centre Health and Technology, University of South-Eastern Norway, Drammen, Norway; 5 Norwegian National Advisory Unit on Learning and Mastery in Health, Oslo University Hospital, Oslo, Norway; 6 Sunnaas Rehabilitation Hospital, Bjørnemyr, Norway; 7 Department of Clinical and Health Psychology, University of Florida, Gainesville, Florida USA; 8 Department of Anesthesiology and Pain Medicine, School of Medicine, University of Washington, Seattle, Washington USA; 9 Department of Psychology, Health & Technology, University of Twente, Enschede, The Netherlands; 10 Department of Psychiatry & Psychology, Mayo Clinic, Rochester, MN USA; 11 Department of Pain Management and Research, Oslo University Hospital, Oslo, Norway

**Keywords:** Chronic pain, Health personnel, Health services, Telemedicine, eHealth, Qualitative research

## Abstract

**Background:**

Chronic pain constitutes a significant burden for the individuals affected, and is a frequent reason why patients seek health care services. While in-person psychosocial interventions can be of support to people living with chronic pain, such interventions are not always accessible. eHealth interventions may provide greater accessibility, but the evidence and use of digital self-management solutions for chronic pain are still limited and the lack of health care provider input in the development process of such solutions a concern. Therefore, the aim of the current study was to investigate health care providers’ experiences of treating patients with chronic pain, their attitudes towards, and use of, digital solutions in pain management, and their suggestions for content and design elements for a potential digital pain self-management intervention.

**Methods:**

Twelve health care providers representing a variety of health care disciplines participated in semi-structured interviews. The interviews were analyzed using thematic analysis.

**Results:**

The material was analyzed into three main themes: [1] Patients with chronic pain and their current use of the health care services, [2] Health care providers’ own motivation and impression of patient prerequisites for use of digital self-management interventions, and [3] Suggestions for content and design elements in a digital self-management intervention for people living with chronic pain. The challenges faced by patients living with chronic pain were described as numerous. Despite interest and positive attitudes, few of the health care providers had used or recommended eHealth solutions to their patients. A range of potential content and functionality elements were identified, including aspects of motivation and engagement and providers also emphasized the importance of easy access and positive, personal content to support existing treatment.

**Conclusions:**

This study offers insights into health care providers’ considerations for the potential of digital self-management interventions supporting patients living with chronic pain. Findings indicate the need for change and a more comprehensive treatment approach to pain management. eHealth solutions may contribute to such change, and providers pointed to a need for health care provider involvement, timely support and follow-up as important factors for integrating digital pain self-management interventions into clinical care.

**Trial registration:**

ClinicalTrials.gov: NCT03705104

**Supplementary Information:**

The online version contains supplementary material available at 10.1186/s12913-021-06278-7.

## Background

Chronic pain constitutes a significant burden for the individuals affected as well as for society at large. It is estimated that as much as 30% of adults may suffer from chronic pain (i.e. defined as pain lasting more than 3 months) [1, 2]. In addition to physical symptoms, chronic pain is often associated with psychological challenges, including emotional distress, symptoms of anxiety and depression, and an overall reduced quality of life [3–5]. Chronic pain also has considerable socioeconomic impact, often leading to sick leave and unemployment [1, 2, 6]. Due to the complex nature of chronic pain and its multifaceted impact on a person’s life, patients with chronic pain need follow-up from a wide range of health care providers [7], and a strong association has also been seen between chronic pain and use of health care services [1, 8–10].

Substantial evidence supports an interdisciplinary and biopsychosocial approach as the most effective and cost-efficient practice [3, 7, 8, 11, 12]. However, this type of comprehensive approach is not yet widely available [8, 13]. Many patients are only seeing their general practitioner and few receive treatment from pain specialists [1, 13]. Long waitlists to see a specialist, limited pain expertise among health care providers, and the scarcity of resources and services available to people with chronic pain are also challenging [1, 8, 13–16]. The need for change in health care services’ approach to pain management is evident.

One aspect that may aid in the process towards a more comprehensive approach to pain management is an enhanced availability and facilitation of self-management support. The goal of self-management in chronic illness is to provide patients with skills and strategies that may help them cope when aiming to manage medical, emotional, cognitive and behavioral facets accompanying their illness [17, 18]. Current international clinical guidelines recommend self-management interventions as a part of pain management [19], and helping patients gain insight into the importance of self-management and teaching them self-management skills can help reduce physical and psychological symptoms and health care use [20]. Psychosocial interventions focusing on self-management in chronic pain often include cognitive behavioral therapy (CBT) [21] and/or acceptance and commitment therapy (ACT) [22] and have been linked to reduced symptoms of distress, anxiety and depression; reduced pain interference; and increased physical functioning, self-efficacy and quality of life for patients living with chronic pain [20, 23–26]. Such treatment is not widely accessible however, with barriers including limited availability of such interventions, geographical distance, costs/insurance coverage, physical barriers and personal comfort challenges [8, 27].

eHealth, defined as “*the use of technology to support health, well-being and healthcare*” ( [28], p7) may provide a way to improve accessibility and outreach of such interventions, and evidence of the potential positive impact of eHealth interventions in chronic pain is growing [29–34]. Such eHealth interventions could potentially also reduce waitlists, enhance treatment durability and possibly also introduce more cost-effective treatment options [31, 32, 35, 36]. However, there is still a gap between the number of digital “pain management self-help” programs available, and the actual number of such interventions that are evidence-based and shown to be effective [37, 38]. Also, low intervention adherence and high attrition rates have emerged as a challenge for eHealth interventions [29, 39], potentially indicating lack of theoretical rationale, lack of focus on potential barriers and facilitators for use and implementation, and a lack of patient and health care provider input in the development process [29, 36–38, 40–44].

A recent study partly addressed some of these issues, identifying patients’ needs and requirements for a chronic pain self-management intervention [45]. For eHealth interventions to be sustainable, however, health care provider input in the development process, and clinician acceptance, are also key factors [44, 46]. A review showed that out of 279 identified pain self-management applications, only 8.2% included health care providers in the development process [42]. Studies have shown that health care providers are generally positive towards digital health applications for chronic diseases, including chronic pain [47–50]. Despite interest in and positive attitudes towards eHealth solutions, few health care providers recommend the use of applications (apps) or digital solutions to their patients [48, 51], and the overall use of eHealth solutions in health care services is limited [48, 49]. Given the apparent lack of theoretical rationale and inclusion of a comprehensive pain management approach in existing pain self-management apps so far [42], consulting health care providers in eHealth intervention design and development appears essential. Including health care providers in such development processes may also contribute to a better understanding of how to address the significant adherence/attrition issue with eHealth interventions [29, 39] another important aspect in the pursuit of overcoming the many barriers to eHealth pain management use [50].

The aim of the current study was to investigate health care providers’ experiences of treating patients with chronic pain, their attitudes towards, and use of, digital solutions in pain management, and their suggestions for content and design elements for a potential digital self-management intervention for people living with chronic pain.

## Methods

### Study design and participants

This study is part of a larger project aiming to design, develop, pilot test and examine the effectiveness of a digital self-management intervention for patients living with chronic pain in Norway. Initial processes include interviews with patients, spouses and health care providers to inform the process of designing and developing the intervention. Interviews with patients and spouses have been reported elsewhere [45]. The research team (i.e. authors of the current paper) consisted of individuals with background from clinical practice treating patients with chronic pain as well as individuals with competence in eHealth development and research, a combination suitable to conduct the current study.

The study utilized a qualitative and exploratory research design, enabling an extensive exploration of the research topic from a variety of angles [52]. Data were collected through individual interviews. The study participants were intentionally selected (i.e., purposive sampling) [53] to ensure insight from a heterogeneous sample of health care providers working with patients with chronic pain. Participants representing a variety of health care disciplines within chronic pain management were included in this study. Potential participants were identified and contacted based on professional acquaintances and recommendations from participants already included in the study (i.e., snowball sampling) [53]. A member of the research team (KH – clinical psychologist working with patients with chronic pain in a pain clinic) contacted the potential participants by telephone or e-mail. All contacted health care providers agreed to participate. Inclusion criteria were: Working as licensed health care provider and offering treatment, care and/or follow-up to patients with chronic pain. The study was approved by the Oslo University Hospital department for data protection and information security (i.e., Institutional Review Board) (approval number: 2017/6697). Written informed consent was obtained from all participants.

### Data collection

Information about age, professional background, postgraduate training and years of experience working with people with chronic pain was collected using a demographic form. The interviews were conducted using a semi-structured interview guide developed for this study, containing questions about the type of patients seen by the health care providers, the treatment and follow-up offered, patient-related collaboration with colleagues in other parts of the health care services, and their experiences and use of digital solutions and interventions (see Additional file 1). Attitudes towards and suggestions for, a potential digital solution for self-management of chronic pain were also explored. The interviews were conducted face-to-face by a member of the research team (KH) at the participants’ workplace and lasted 20–110 min (median 74 min). The first interview was conducted by KH and the first author together and acted as a pilot interview. As this interview captured issues of interest and prompted no changes in the interview guide, data from the pilot interview were included in the study. All interviews were audio-recorded and transcribed verbatim.

### Data analysis

Transcripts were analyzed using thematic analysis [54]. In the first step of the analysis, three of the authors (CV, ILS and HE) listened to the recordings, read through the transcripts and individually took notes to familiarize themselves with the content. Next, CV and ILS deductively coded the transcripts into three pre-defined overarching themes based on the interview guide and using the software program NVivo version 11 (QSR International, Victoria, Australia): 1) Provider identified patient characteristics, 2) Treatment and follow-up offered to patients with chronic pain, and 3) Interviewee’s experience using technology, and the identified needs and requirements for a future digital chronic pain self-management intervention. Data that could not be coded into the predefined themes were assigned new themes. Identifying patterns in the material, themes and sub-themes were created, and the transcripts were coded into these. This way, the coding process was deductive based on the interview guide, as well as inductive in terms of how new themes were created if the transcripts could not fit into the predefined themes. The transcripts were reviewed for content relevant for the aim of the study, and systematically coded into the themes. Each theme was then re-examined, identifying variations and similarities within the themes. The themes and sub-themes were then discussed and reviewed, renamed and re-arranged in an iterative process into a final structure, which at times differed from the initial pre-defined overarching themes. The pre-defined categories were changed in the final presentation of themes in the findings.

Researcher reflexivity regarding the research team’s influence on the study results [55, 56] was addressed to increase trustworthiness. This was done through reflections and discussions between several of the authors (CV, ILS, HE, LSN) regarding the study aim, the research gap the study was able to fill, as well as analysis and interpretation of data. The analysis was conducted in an iterative process that involved several researchers and results were finalized following several rounds of discussions in the research team.

## Results

### Participant information

In total, 12 health care providers (5 men and 7 women) participated in the interviews. They represented the following health professions: psychologist (*n* = 1), general practitioner (*n* = 3), registered nurse (*n =* 1), occupational therapist (*n =* 1), physical therapist (*n =* 3), social worker (*n =* 1), pain specialist (medical doctor - MD) (*n =* 1) and psychiatrist (*n =* 1). Participants were between 30 and 70 years old (median 57). They had an average of 26 years of clinical experience (range 2.5–43) and on average 17 years of experience treating patients with chronic pain (range 2.5–33).

### Overview

**Fig. 1:**
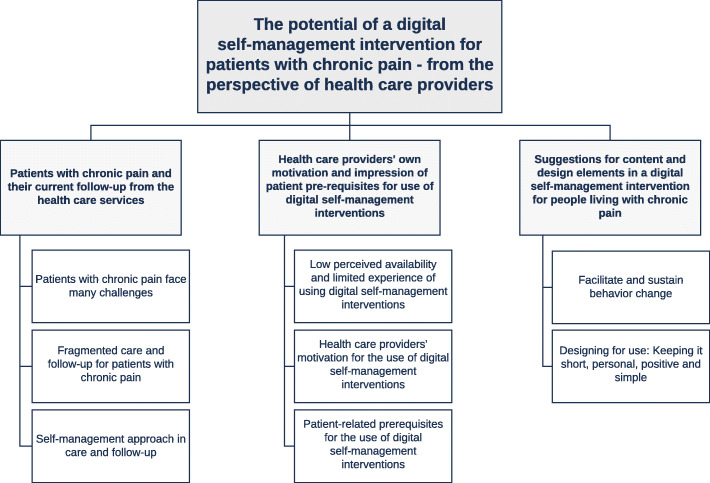
Overview of the main themes and sub-themes

The material was analyzed into three main themes: (1) Patients with chronic pain and their current use of the health care services, (2) Health care providers’ own motivation and impression of patient prerequisites for use of digital self-management interventions, and (3) Suggestions for content and design elements in a digital self-management intervention. Main themes and sub-themes are illustrated in Fig. 1.

### Patients with chronic pain and their current follow-up from the health care services

The health care providers in this study had many reflections regarding the characteristics of patients living with chronic pain, how the health care services provided patient care and follow-up, and the providers’ role in the self-management process. These aspects are described in the following sections.

#### Patients with chronic pain face many challenges

The health care providers had experience treating patients with a wide variety of chronic pain conditions as defined in ICD-11 [57], including pain related to fibromyalgia, arthritis, arthrosis, psoriasis, multiple sclerosis, myalgic encephalopathy, neuropathy, accidents, injuries, surgeries, and unspecific musculoskeletal pain (e.g., back and neck pain). They acknowledged that living with chronic pain must be very challenging, describing patients as experiencing challenges related to cognitive, emotional, psychosocial and economic factors. Issues such as anxiety, depression and psychosomatic disorders were discussed, and many patients were described to struggle with worries, fear and insecurity about their condition and the future. Negative thinking, rumination, sadness, hopelessness, feeling unsuccessful and having limited experience of self-management were also described as common. One of the health care providers said:

It’s kind of these negative thoughts and the rumination and sense of failure, feeling inadequate, and sadness about the situation. (Occupational therapist)

The providers also stated that patients often struggle with sleep challenges, fatigue and activity-pacing issues. Many patients frequently expend almost all their energy on everyday activities, often including supporting others. The providers also described many patients with chronic pain as carrying significant burdens in other aspects of their life. One of the health care providers said:

When the pain is yet another burden on top of many others, they have an even bigger issue dealing with it. [...] It’s not just the pain itself, but perhaps the sum of all their burdens that is the challenge. (Psychiatrist)

Providers also described many patients as expressing a need to be seen, heard, believed and respected by their family, friends, health care providers and even themselves. Many patients had communicated that they feel like they are being sent back and forth in the system, that no one really takes charge of what is going on with them, and that they have no stable health care provider relationship to support them in dealing with the situation. According to one health care provider:

What may be characteristic for all of them is that they feel like they can’t get help, anywhere. [...] They feel like nobody can fully grasp and understand their situation. (General practitioner)

The health care providers emphasized the importance of patients taking an active role in their own pain management process, albeit acknowledging that many patients find this difficult. They stated that this is often because the patients have not yet accepted their situation and are instead continuously searching for diagnoses, causes, and possible solutions, new treatments or preferably a cure. The providers acknowledged that when they, or other health care providers, keep referring them around in the system, it may be more difficult for patients to take charge of their own situation on a daily basis. One provider stated:

It’s always an advantage to try to be as clear as possible about as many things as possible and perhaps also end the evaluations, and think this has gone on long enough and now here we are. We have to draw the line, you’re well examined and evaluated, so now we have to look towards how to handle this as we move ahead, and sort of change course. (Physical therapist)

Providers also described lack of knowledge and difficulties finding trustworthy information as challenges for many patients, despite having lived with chronic pain for a long time. Additional comments included:

I think many of them need to know more about their pain, need more information. And some need tools to put that knowledge to use. (Social worker)

It’s a jungle for them to figure out which information about coping or treatment is reliable. (Psychologist)

#### Fragmented care and follow-up for patients with chronic pain

Interviews with the health care providers in this study indicated that only a few of the existing services for patients with chronic pain can be referred to as cross-professional and interdisciplinary. Pain clinics may be described as such, but the providers stated that health care providers usually collaborate mainly with providers of their own profession, and most often simply work alone. As a result, no one truly has a full overview of the treatment and follow-up of each patient and subsequently care becomes fragmented. One health care provider explained:

They’re missing the whole picture, that’s what is always missing. Especially when health care providers don’t agree, I mean when the providers have a discord in interpretations or explanations. And I always find that challenging, as it puts the patient in difficult situations, where the patient is trapped between different opinions. (Physical therapist)

Many of the health care providers related having tried to establish contact and collaboration with providers in other health care specialties, but with limited success. Even if they found the collaboration between different health care services suboptimal, they acknowledged the other professionals’ competence, and as such seemed to be accepting fragmented care and follow-up. One health care provider said:

I really try to let the specialists handle their own tasks, while I handle mine, and then just get specific feedback from them about what I should be doing as general practitioner. Because there’s no way I can be good at everything, so I have to acknowledge my own limitations, even though that leads to lots of referrals and consults. (General practitioner)

The providers also stated that neither the health care providers nor the patients have full knowledge as to which health care services are available (including self-management support). Some services are not affordable for some patients, which means that some patients utilize a variety of services while others only use a few. One provider stated:

There are probably some [patients] who don’t have many treatment options, and then there are some that have a lot of treatment options. (Psychologist)

#### Self-management approach in care and follow-up

The interviews indicated a large variety in the extent to which the health care providers emphasize psychosocial aspects in their clinical work with patients. The psychiatrist in this study said:

I feel that my challenge can be to convince people that it’s useful to work with the psychological aspects of pain, even if they’re psychologically healthy [...] Living with pain does something to us psychologically, and that how we relate to that psychologically means quite a bit for quality of life as well as even the actual pain levels [...] How much you emphasize this issue varies, and also to what degree the patient feels it’s okay to bring up that type of problem. (Psychiatrist)

About half of the health care providers clearly stated that they include psychosocial aspects when treating patients with chronic pain. Some of them also expressed a wish for a greater focus on these aspects from other providers in the health care services. One health care provider explained:

I wish there was more focus on social aspects. The biopsychosocial insight that I feel a lot of people claim to have. But in reality it’s often a biopsychological insight. Or it doesn’t go beyond that. Or it’s actually a bio-bio-bio insight, and then we can talk with the psychologist if we feel like it. That kind of thing. That the pharmaceuticals often get top priority, taking center stage. And then everything else gets tacked on around the medical part, around the physician’s expertise. But you could benefit from including more aspects of life in this. (Social worker)

Independent of treatment approach, all the health care providers in this study emphasized the importance of patients taking the lead and taking an active role in the pain management process. A general practitioner said:

I usually say, and I say this very often, what we can do is very little, what you can do is tremendous. The physical therapists can also do very little. They can help you some, give you some good advice etc., but you’re the one who has to do the work. And we can keep trying for a hundred years, but we’ll never move forward if you don’t do it. Sometimes that message gets through, sometimes they think I’m a lousy doctor who couldn’t do more for them. (General practitioner)

Many of the health care providers in the current study assigned patients homework between treatment visits, emphasizing the importance of the patients’ own efforts to live as well as possible with the pain. However, they stressed that the amount of homework must be adapted to the individual patients’ abilities and motivation, as the patients are not always able to complete the homework given.

### Health care providers’ own motivation and impression of patient prerequisites for use of digital self-management interventions

The interviews in this study provided insight into the health care providers’ reflections regarding their own motivation as well as what they believed concerning patients’ attitudes and prerequisites for the use of digital pain management interventions. These aspects are described in the following sections.

#### Low perceived availability and limited experience of using digital self-management interventions

Despite acknowledging the growing use of digital solutions within health care services and society in general, few of the health care providers in this study had recommended or used digital solutions together with patients. Those who had used digital solutions had used apps and websites for yoga, mindfulness and CBT. Only one of the health care providers had used what was referred to as a pain management application. This app was in a foreign language, and therefore not suitable for all patients due to the language barrier. Another health care provider had used a migraine app for personal use. About digital self-management interventions, an approach shared by many of the providers was described as:

I’ve never been introduced to one, and I’ve never had any recommendations for apps. (Pain specialist (MD)

#### Health care providers’ motivation for the use of digital self-management interventions

The motivation for making use of digital self-management interventions ranged from strong to neutral among the health care providers in the current study. No clearly negative attitudes were expressed. The providers who were positive underlined that digitalization is the future and has the potential to reach many patients with evidence-based interventions in a cost-effective manner. One of the health care providers said about digital self-management interventions:

They’re available, and I think that they can reach a lot of people, and if I think cost-benefit, and that many of them [patients] probably need just a little bit input to make major changes, it could mean a lot. Maybe I’m thinking especially about people who can’t afford much, and where resources are scarce, or they have other family [pressures], so they can take the digital solution with them whenever or wherever it’s convenient, and maybe those most in need of something, and being able to giving it to everyone, that makes it a bit meaningful, and that it can be evidence-based and help them find their way to something useful, in the jungle of all kinds of things, I think that’s good. (Psychologist)

The providers who were more skeptical towards using digital self-management interventions attributed their reluctance to personal preference, more advanced age and that meeting patients in person is important for treatment effectiveness. According to one of the health care providers:

If everything is left to itself and an app, then they kind of lose the dimension of humanity that we usually have. If it's just an app and nothing else, then it might be a little sparse [...] Chronic pain has so many dimensions, including loneliness, which might not necessarily be cured with an app. (Physical therapist)

Another provider described reluctance related to personal preference:

I have problems getting started with things like that, I think it’s a bit irritating. It has to do with me personally not liking to use them. [...] I have to struggle to motivate myself to start using them. My belief is that they’re so omnipresent nowadays that it’s old-fashioned and foolish to not want to use them, but it’s still important not let ourselves be persuaded to streamline and drop face-to-face contact. (Psychiatrist)

Some of the health care providers described a digital intervention as potentially useful for them as health personnel, viewing it as an additional source for knowledge and education that might improve the quality of the treatment for their patients. One health care provider said:

They’re at least as useful for the therapist, as an entryway to offer the patient something better, something that in the long run may help the patient become more independent and break out of these treatment cycles that are often useless, and costly for society, and that frequently aren’t very well documented either. (Physical therapist)

#### Patient-related prerequisites for the use of digital self-management interventions

Regarding potential usefulness of digital self-management interventions for patients with chronic pain, health care providers stated that a digital intervention would likely be particularly useful for patients who are motivated to work with their own situation. Referring to patients who are already using self-management interventions, one of the providers, in response to the question of what characterizes patients who are able to use digital interventions, stated:

They’re capable people, with such a drive to get well, they want to get well, they want to do things to help themselves, they try a lot of different things, and the prognosis for some of them is better, too. They say, you know what? That’s not how I want it to be, I want to do something. And they also ask, what can I do. (General practitioner)

One of the health care providers also stated that there may be many reasons why patients might prefer digital over in-person interventions, including practical issues or aspects related to shame or discomfort.

The health care providers in the current study also had some thoughts about which patients would not benefit from digital self-management interventions. For example, cognitive impairment and advanced age were mentioned by several of the health care providers as potential barriers for use. One of them said:

The older patients I’ve been treating for so many years, so when I’m supposed to start using apps, it will be difficult for them to understand. I mean, they still can’t even turn in computerized sickness leave forms. (General practitioner)

Patients with cultural or linguistic barriers, low eHealth literacy or limited resources were also mentioned as potentially facing challenges with eHealth use. The health care providers also mentioned low motivation or lack of motivation, either in general or due to conditions such as depression or persistent pain, as potentially limiting usefulness. As expressed by one health care provider:

Most of them are so burdened with their lives and their pain that they don’t have the energy to get involved in such [apps] either. (General practitioner)

One health care provider also mentioned that the use of digital interventions requires equipment that not everyone can afford.

### Suggestions for content and design elements in a digital self-management intervention for people living with chronic pain

The health care providers shared multiple ideas and suggestions for the content, functionality and design of a potential digital self-management intervention for chronic pain. They emphasized the need for an easily accessible, holistic and comprehensive solution that can facilitate and sustain treatment effects and behavior change for patients with chronic pain. This input is described in the following sections.

#### Facilitate and sustain behavior change

With chronic pain issues being complex and often difficult to alleviate, the health care providers emphasized the importance of supporting patients in bringing about behavior change, and that such change could potentially be facilitated through help in terms of changing focus, habits and daily routines. They stated that this would require regularity in efforts to bring about change if change should be maintained beyond the course of the treatment. One health care provider said:

I think change is really difficult, to turn things around and balance change, connected to one’s inner drive or being active in one’s own life [...] How to bring about a change that can be extended beyond [the treatment] is completed, because I sense that they get pretty involved and inspired and so on, and then I’m not quite sure how long that lasts. (Psychologist)

The health care providers described digital interventions as potentially providing patients with access to evidence-based and trustworthy information as well as access to practical exercises and/or illustrative examples over time. The psychologist stated:

It has to offer them something specifically linked to how they can actually make that change, some information related to that. And other elements can probably be included as well, so there’s like a buffer, [...] which means that there has to be some specific exercises and some kind of range, so that activity can be seen over time. Like OK, now you can try this until the next time, and then we’ll see how you think things have been going, kind of like a way to show a structured way of working with change, for example. [...] They take their phones along everywhere which would provide really easy access. (Psychologist)

Many providers also mentioned the potential of using a digital intervention as a supplement to the treatment provided by them. One provider stated:

This would be a really useful supplement, I mean you could use in treatment and it could strengthen my message. It could also be something they could work with between consultations, and if there were a few homework exercises in the app, stuff they could work on as a supplement, that could be useful. [...] It could kind of reinforce what we did here, that could be very useful. (Physical therapist)

The health care providers described multiple topics that could be included in the intervention, ranging from information about pain physiology, and more specifically related to specific diagnoses, health promotion (e.g., information about sleep, nutrition and physical activity), information about medications and treatment, and available support and welfare resources. Links to web pages and information about psychological challenges related to chronic pain were also suggested, as were education about coping skills. Relaxation and yoga exercises were mentioned as useful exercises. Many of the health care providers also brought up the potential usefulness of patients making notes/registering variables such as pain, physical activity and sleep, so that the patients could become more aware of their activities and behavior, and the potential associations to their experiences of pain. Some also suggested that patients could list their pain medication and types of treatment they had tried, so that patients could share these registrations with their health care provider, or get personal feedback and recommendations (automatically or from their health care provider) in the digital program. One of the health care providers stated:

If they have a goal to, let’s say, exercise twice a week, in order to manage that, for half an hour, if that’s their goal, then there’s something about maybe having an activity plan and a log where you actually note, well, Monday this and that, exercised half an hour, and then maybe a small section where it said; “went really well” or something, or “this feels great” or that you can write down something about what it was like, maybe some have options for “check off” this or that. [...] In itself, it would be a way to cope to see that, wow, I accomplished that twice this week, and I did it last week too, yes. [...] One of those smileys could pop up, like nice work, you did it again. (Physical therapist)

The majority of the health care providers described the potential for contact between patients and health care providers as important and necessary. They emphasized the possible challenges of keeping patients motivated, and the importance of being able to follow up on patients’ progress and goals. Several providers stressed the importance of providers and patients practicing using the intervention together during the initial phase. Some also mentioned the possibility of patients using the digital solution to complete forms and questionnaires, giving health care providers direct access to patients’ answers and results. All health care providers agreed that contact between health care personnel and patients would be useful, especially for the patients. Many, particularly the physicians and psychologist, did however express doubts about how this could work for them as health care providers with very limited time available.

#### Designing for use: keeping it short, personal, positive and simple

All health care providers emphasized the importance of user-friendly and intuitive design, without too much information or text on each screen, taking into account the concentration issues that many patients with chronic pain have to cope with. Some pointed out that it was important not to use too many colors, as many patients struggle with hypersensitivity to light. Another suggestion was that instead of having patients respond to questions in writing, multiple-choices or smileys options could be available, making exercises and assessments more effective and easy to complete. Many health care providers thought using videos or illustrations could be good ways to present content. Several did however state that shorter texts likely would be good for this user group, as that would allow patients to read the content as many times as necessary, again emphasizing the concentration challenges many patients face. Several providers also suggested adding push notifications, reminding people to work with the content and exercises on a daily basis, as that would promote incorporation of new knowledge and possibly new habits.

The health care providers also emphasized the importance of conveying hope, and shifting the focus away from the pain and instead towards resources, strengths and coping. One health care provider said:

It should be something that conveys hope and useful coping, and not lingering in misery; the strength perspective should be in place. [...] That there is hope and an expectation for improvements, and that it can be a support for good coping strategies. (Registered nurse)

To accommodate this, one health care provider said:

It should explain to people that falling back into old patterns is perfectly normal, but that they have to establish a new pattern, and that’s not easy. Let them know it’s okay to slip up, it doesn’t always have to be perfect. (Physical therapist)

The importance of using positive, non-offensive and motivating language and design was also addressed, focusing on usability. Several health care providers stated that it was important to avoid too many medical terms, or overuse of the word “chronic pain”. One health care provider said:

It has to be enjoyable. Something that can offer something [...] not exactly joy, but that somehow there’s hope. Whatever information you give, there always has to be a degree of hope. The hopelessness has enough room already. (Physical therapist)

Some of the health care providers emphasized the importance of adjusting the intervention to the individual patient, letting the users choose their own personal goals and which content and exercises to perform, providing them with personal reminders based on their own recordings.

I think it’s fairly important [...] that it can be tailored a bit. Even if it’s only the first time you make an assessment that you say, OK, what are your goals. And then they mention four things they think are important, and those are the ones that end up on the main screen. Just something that makes them feel like “this is my tool.” (Social worker)

One of the health care providers did however state that this might be difficult, as patients do not necessarily know what is important to them.

Many providers also stated that one of the biggest risks and barriers for use would be that the intervention becomes too large and extensive. Some also stressed that it is important not to overwhelm patients by giving them too many alternatives to choose from. One also suggested building the intervention stepwise, adding more and more content over time.

Finally, health care providers emphasized the importance of quick and easy access to the intervention, for themselves as health care providers with limited time as well as for the patients. Suggestions included a simple login without a password, and access to the intervention via cell phone rather than a personal computer.

## Discussion

The current study investigated health care providers’ experiences when treating patients with chronic pain, and examined their input related to the potential of a digital self-management intervention supporting existing health care services in pain treatment. The health care providers interviewed in this study described a range of challenges faced by patients related to living with chronic pain, and portrayed challenges with apparent fragmented health care and disparities in collaborations between health care services and disciplines. The providers emphasized the need for support in facilitating self-management and persistent change for patients with chronic pain. They appreciated the potential of a digital solution in this process, but also described the need for careful attention to how such a solution was designed, developed, presented and implemented into health care delivery and use. A range of content and functionality elements was suggested for inclusion in a digital intervention, and the providers particularly pointed to the need for trustworthy information and exercises focusing on coping, awareness and behavior change. The health care providers further emphasized the importance of the intervention being easily accessible (i.e., easy to use and gain access to), motivating, positive and personal. The following sections discuss results in light of how eHealth self-management solutions might support pain management, issues raised, and how to facilitate implementation of digital interventions into health care services.

### The potential of eHealth self-management interventions for chronic pain

The health care providers in the current study described pain management as challenging, and emphasized the need for support in facilitating self-management, hope and persistent change for patients with chronic pain. While the providers were positive towards the potential of a digital solution in support of patients with chronic pain, they also raised a number of aspects that should be considered for such a solution. The following elaborates on these aspects and how an eHealth solution can support patients and health care providers in the pain management process.

#### Supporting self-management and behavioral change

Studies have shown that providing patients with self-management skills can reduce the experience of pain while improving overall mental health and quality of life [18, 20, 58]. This was also emphasized by patients in a study examining patients’ needs and requirements of an eHealth self-management intervention for chronic pain [45]. The health care providers in the current study supported this notion, but described teaching patients the skills necessary for this process as challenging. In particular, health care providers reported seeing how patients had difficulty putting knowledge to use in their everyday lives, thereby also having difficulty achieving the behavior change needed to change habits and improve coping. With this in mind, the health care providers in the current study expressed a need for more knowledge and specific exercises to share with their patients, and for such information to also be available in a potential digital self-management program for chronic pain.

A number of studies have illustrated the potential of eHealth solutions in promoting self-management skills and behavior change for patients with chronic pain [29, 34, 40, 59, 60]. However, despite significant potential, the scientific literature has pointed to the lack of comprehensive digital pain management solutions, stating that eHealth solutions often only include subsets of the available pain management components and functionalities. For instance, a systematic review reported that 62.3% of the pain management apps examined offered self-monitoring and 24.1% pain education, but only 13.6% offered both [61]. Two other reviews had similar findings, showing that the examined apps included only few self-management functionalities [42, 51]. This is also consistent with the findings from the current study, where providers emphasized the need to include multiple topics and functionalities in a potential pain self-management intervention.

Such a multicomponent pain self-management solution can potentially support patients in their efforts to incorporate new knowledge into daily practice, to make the needed change (e.g., habits, behavior, thoughts, emotions) [20], and also have the potential to provide particular skills needed, for example related to communication, social support and assertiveness (i.e., expressing specific needs and requirements). The current findings are in line with existing research showing how treatment approaches vary, how chronic pain health care may be perceived as fragmented, and pointing to a need for more cohesive and comprehensive pain management [1, 13, 45, 62].

#### Promoting motivation and engagement

Finding the energy and motivation to acquire new skills and engage in new coping strategies, as well as maintaining such change over time, were acknowledged by the health care providers in the current study as demanding. This has also been reported by previous studies [63, 64]. However, providing patients with educational information and new skills, delivered through frequent input, in everyday life and in-between regular treatment, could support such change. In an eHealth solution, the use of design elements and functionality could support such delivery and potentially motivate patients for continued and prolonged use [39]. In the current study, health care providers emphasized the importance of focusing on resources and coping skills to enhance motivation and engagement. For an eHealth solution, this included the use of supportive language and an enjoyable design, potentially promoting a feeling of hope to motivate for persistent and continued use. This is in line with existing research showing hope to be an essential motivator for self-care activities [65]. Studies have also shown that the use of persuasive design and gamifying elements in eHealth can promote user engagement [39, 66]. The use of personalization and tailoring in eHealth have also been associated with promoting user engagement [36, 66], and some of the providers in the current study suggested personalization of the intervention to enhance patient engagement. Personalization and/or options for tailoring may also be relevant to potential homework assignments (e.g., CBT based), as some patients living with chronic pain, depending on situation, may struggle to complete homework. The health care providers also emphasized the importance of reminders (i.e., digital push notifications), so that patients would remember to do and practice content, an essential part of acquiring and adapting to new skills and knowledge. The idea of push notifications is supported by research, with links to increased engagement with eHealth solutions [67]. The suggestions made by health care providers in the current study, aiming to improve motivation and engagement were all in line with suggestions previously made by patients with chronic pain and their spouses [45].

The majority of the health care providers in the current study also pointed to health care provider involvement and options for digital patient-provider communication as potential factors of importance to motivate patients to use such a tool and support them in the self-management process. Health care provider involvement could also help prevent patients from feeling as if they are personally responsible for “succeeding” with the intervention. As such, health care provider involvement could limit or prevent potential self-blame and otherwise negative impact should patients struggle to cope, despite engaging in digital self-management. Other studies have also found that guided intervention and blended care can facilitate eHealth acceptance and promote engagement [59, 68]. The health care providers in the present study describe how patients with chronic pain commonly present with complex issues, including lack of self-worth, loneliness and depression. They suggest that for some patients such underlying issues must be addressed if motivation to use and benefit from eHealth intervention is to be expected. These results may argue for blended care where the eHealth intervention could supplement communication with the health care provider where active listening and acknowledgement of existential concerns might be essential [69]. Adding guidance and personal feedback is also a way of tailoring the intervention [70], as suggested in the current study. Guidance and personal feedback or follow-up could potentially also help address the attrition/adherence challenges associated with such interventions [29, 39, 59, 68]. However, human involvement and support in the form of guidance also raises eHealth intervention costs. Establishing when, if and how interventions should be supplemented by such support is therefore important [71, 72], and identifying ways to provide some other form of guidance (i.e., built into the eHealth program) may be a necessity [73].

#### Addressing individual challenges for eHealth use

A number of individual barriers for patients’ use of eHealth were addressed by the health care providers in the current study. These included cognitive impairment, advanced age, low motivation, limited resources, low eHealth literacy and linguistic and cultural barriers, all of which are in line with existing research [74–76]. These are important aspects to take into account when developing eHealth solutions and offering them to patients, otherwise the digital divide will continue to increase [74]. For example, the use of eHealth can be encouraged through improved access to devices and the Internet to people with limited resources [74, 75]. Also, the need to match eHealth technology to patients’ general and health-literacy levels have been described to be important to help establish effective eHealth interventions [77]. Regarding advanced age as a barrier for eHealth use, one study has found that older persons with chronic pain were positive towards the use of eHealth [78]. However, several studies have pointed to the need for targeted training and information to these patients regarding how the eHealth solution can be valuable for them [74, 78].

### Integration of digital self-management interventions for chronic pain into clinical practice

The health care providers in this study showed overall positive attitudes towards the potential of eHealth, but in line with existing research [48, 49], they had limited personal experience using eHealth solutions in pain treatment. This brings the attention to the importance of considering certain aspects when implementing digital interventions for pain self-management into clinical practice.

#### Urgent need for change as an implementation driver

A number of studies, including the current one, have identified many barriers that may hinder successful integration of digital pain management interventions into clinical practice. This includes limited exposure to or awareness of eHealth solutions, provider concerns about increased workload, provider and patient technological challenges, intervention content not customized to the patient group, and skepticism towards replacing in-person with digital interventions [76, 79]. However, if these issues can be resolved, many barriers have counterparts that act as facilitators in terms motivation, resource availability and willingness to integrate eHealth into care [76, 80]. In the current study, the health care providers expressed a need for change in the care and follow-up of patients with chronic pain. They described chronic pain as complex, sometimes involving multiple conditions, the care and follow-up as often fragmented and suboptimal, and their own competence with chronic pain as somewhat limited. As such, the providers identified a gap in their own abilities to offer optimal care and follow-up for the patients, and supported the idea of eHealth interventions as a supplement to existing care that could potentially contribute to closing this gap. The issues raised indicate an urgent need for practice change, which could also act as a facilitator and incentive for eHealth implementation [44, 81].

#### Stakeholder engagement in the design and development process

The current study sought to address the issue with lack of health care provider involvement in the development of eHealth interventions [29, 42, 82]. eHealth solutions have often been developed in response to technological innovation and technical possibilities, without including the needs and requirements of potential users (i.e., patients and health care providers) in the development process [31, 37, 41, 42]. This lack of stakeholder input in the development of eHealth solutions may at least partly explain the low adherence and high attrition rates associated with the use of such interventions [29, 39, 59]. Including health care providers in the planning and execution process of implementing a given eHealth intervention into a particular context is imperative [83–85].

The reported limited availability of time and resources in the clinical setting is a well-known challenge [8, 64], further underlining the need for health care provider input in the design and development process of eHealth interventions to avoid development of interventions that cannot be used due to time and other constraints. The current study also indicated that as patients with chronic pain receive treatment from a variety of health care services, with each specialty utilizing their own treatment approach, representatives from all involved health care treatment types and services should be involved in the design and development process, to ensure that digital self-management interventions fits into the intended health care settings.

#### Need for support when starting to use new eHealth interventions

Although health care providers in the current study appeared generally positive, expressing an urgent need for something that can be of support in the care and follow-up of patients with chronic pain, findings from this study revealed that eHealth solutions are new to many of them. This likely means that many providers will need support and follow-up when starting to use new eHealth interventions, underlining the importance of putting enough effort into the implementation process for such interventions to succeed [85, 86]. In the current study, the perceived need for change seemed to outweigh any skepticism and resistance expressed by the providers towards a new technology. This corresponds with a recent study that concluded that change implementation is more likely to succeed when health care providers are involved, feel prepared and recognize the value and patient related benefits of the change [87].

### Strengths and limitations

The current study has some strengths and limitations that should be addressed. First, the study included only twelve health care providers. However, even though the sample is small, the wide variety of provider disciplines and balanced representation of health care practices included, representing both primary and specialist care, can be considered a strength. Also, the current study aimed to gather rich and varied data to aid in the design and development of an eHealth chronic pain self-management intervention, which means that statements from one health care provider could be as important as statements of the majority. The health care provider interviews were also relatively long in the current study, thus providing in-depth understanding of the health care providers’ point of view. In addition, according to the concept of information power by Malterud (2016) [88], aspects related to the study aim (i.e., neither narrow nor wide but somewhat in between), the sample (i.e., containing a variety of health care providers representing several angles for pain treatment and follow-up), the data (i.e., rich and deep) and the theoretical foundation of the study, the number of participants were considered adequate.

Second, the goal of the study was not to determine whether or not to develop a digital solution for chronic pain self-management, but rather to examine the health care providers’ attitude towards, use of, and suggestions for content and design elements of such an intervention. This could potentially introduce a researcher bias in the way interviews were conducted and findings interpreted. However, the interviews were semi-structured with pre-determined questions, conducted by research personnel not involved in the data analysis. Also, the analyses ensured that all issues and concerns raised by the health care providers towards an eHealth intervention for pain management, in the current study, were identified and subsequently have been described and discussed.

The fact that the health care providers in the current study spoke on behalf of patients with chronic pain, based on their impression of patients’ needs and prerequisites for use of a digital self-management intervention, could also be considered a limitation. However, this was done to explore provider impressions and complement already examined patient perspectives [45].

That health care providers in the current study had limited personal eHealth experience should also be taken into account, particularly regarding potential use and usefulness of digital self-management interventions in chronic pain. Many of the providers could only describe their best guess or impression of how to use self-management interventions in pain management, which does not necessarily correspond to actual use and outcomes when digital self-management interventions are brought into use in clinical practice.

Finally, the participants represent a variety of treatment functions and approaches. Therefore, it is possible that the eHealth solution will be more useful for some health care providers than others. The nature of the current study did not allow for such estimates, however, this would be an interesting and potentially important aspect to explore in future research testing and implementation of eHealth solutions into clinical practice.

### Future implications

The current study provides important input from health care providers treating patients with chronic pain, specifically addressing the potential needs, requirements and use/usability for a digital pain self-management intervention. The results complement findings from a study examining patient identified needs and requirements for a pain self-management eHealth intervention [45]. Together, these studies illustrate important aspects that need to be considered when developing an eHealth self-management intervention for patients with chronic pain. As such, these studies have provided input towards the design and development of such an intervention [73]. Incorporating evidence-based knowledge, theoretical rationale and the stakeholder (i.e., patients and health care providers) voices were sought to accommodate some of the deficiencies identified in existing literature. It should also be acknowledged that in order to implement the multi-component aspects stressed by health care providers in the current study, such interventions could be too complex, potentially overwhelming and demotivating to patients. Future research therefore needs to test the feasibility, usability and accessibility of such interventions, and a feasibility pilot is currently underway from the recent studies. Following feasibility testing and intervention optimization, a randomized controlled trial will follow to establish intervention efficacy.

## Conclusions

The current study offers insight into health care providers’ experiences in treating patients with chronic pain, and their considerations of the potential of digital self-management interventions in chronic pain in support of existing health care services. The results indicate the need for a more comprehensive treatment approach to the self-management of chronic pain and suggest that eHealth self-management interventions may have the potential to positively contribute to pain management. eHealth solutions could supplement the existing health care services, building a bridge between health care disciplines and providing many patients with pain with a source for comprehensive pain management support potentially facilitating self-management. The study does however highlight the necessity for patient and health care provider involvement, representing all parts of the health care services, in the process of designing an eHealth intervention that will fit the intended context.

## Supplementary Information

**Additional file 1.** Semi-structured interview guide for interviews with health care providers

## Data Availability

The datasets generated and analyzed during the current study are not publicly available due to national ethical regulations, but can be available from the corresponding author on specific request, if approved by the hospital department for data protection and information security (i.e., Institutional Review Board) at Oslo University Hospital.

## Abbreviations

ACT: Acceptance and Commitment Therapy
CBT: Cognitive Behavioral Therapy
MD: Medical Doctor
