# Supplementary material for: Health care providers’ experiences of pain management and attitudes towards digitally supported self-management interventions for chronic pain: a qualitative study
Source: BMC Health Serv Res. 2021 Mar 25;21:275. doi: 10.1186/s12913-021-06278-7 (PMC7992849; doi:10.1186/s12913-021-06278-7)
Supplement: Supplementary file 1 — Additional file 1. Semi-structured interview guide for interviews with health care providers [file 12913_2021_6278_MOESM1_ESM.docx]

# Additional file 1

# Semi-structured interview guide for interviews with health care providers

## Introduction

*Study personnel give a brief introduction about the aim of the study and the topics to be discussed in this interview.*

## Your clinical practice and the patients you see

Can you tell me about a typical day at work, the patients you meet and your patient-related collaboration with colleagues in your own organization and in other parts of the health care services? In your experience, which other health care services do the patients utilize?

## Your competence and experience in chronic pain

Can you tell me about your background, competence and experience working with patients with chronic pain, and to which degree you feel that you have the needed skills to meet the challenges the patients bring to the consultations?

## Use of technology in chronic pain patient follow-up

Can you describe your use of digital solutions and interventions in treatment, care and follow-up of patients with chronic pain? What do you perceive as facilitators and barriers to such use? In your experience, which types of digital solutions do the patients use?

## Suggestions for content and design of a potential digital solution for self-management of chronic pain

I would now like to discuss with you your suggestions for the content of a potential future digital solution for self-management of chronic pain. Also, I would like to hear about which design elements you think it is important to include to make the solution user-friendly and attractive to patients with chronic pain.

## Closing remarks

*Thank the participant for the insight shared and time spent during the interview.*
